# Variation in cardiovascular magnetic resonance myocardial contouring: Insights from an international survey

**DOI:** 10.1002/jmri.26689

**Published:** 2019-02-17

**Authors:** William E. Moody, Lucy E. Hudsmith, Ben Holloway, Thomas A. Treibel, Rhodri Davies, Rebecca Kozor, Christian Hamilton‐Craig, Nicola C. Edwards, William M. Bradlow, James C. Moon, Richard P. Steeds, On behalf of the British Society of Cardiovascular Magnetic Resonance and the Australia and New Zealand Working Group for Cardiovascular Magnetic Resonance

**Affiliations:** ^1^ Department of Cardiology, Royal Brompton Hospital Royal Brompton & Harefield NHS Foundation Trust London UK; ^2^ Institute of Cardiovascular Sciences, College of Medical and Dental Sciences University of Birmingham Birmingham UK; ^3^ Department of Cardiology, Queen Elizabeth Hospital University Hospital Birmingham NHS Foundation Trust Birmingham UK; ^4^ Department of Radiology, Queen Elizabeth Hospital University Hospital Birmingham NHS Foundation Trust Birmingham UK; ^5^ Department of Imaging Barts Heart Centre London UK; ^6^ University of Sydney School of Medicine Sydney Australia; ^7^ Prince Charles Hospital University of Queensland and Griffith University Brisbane Australia; ^8^ Auckland City Hospital Auckland New Zealand

## Abstract

**Level of Evidence:** 5

**Technical Efficacy Stage:** 2

J. Magn. Reson. Imaging 2019;50:1336–1338.

To the Editor:

Cardiac magnetic resonance imaging (MRI) is the reference standard for accurately quantifying cardiac volumes, mass, and systolic function. Standardized methodology published almost a decade ago recommended short‐axis steady‐state free precession (SSFP) cine imaging,^1^ which remains the workhorse of most protocols today. Although the Society of Cardiovascular Magnetic Resonance (SCMR) recently acknowledged "moderate variability" in normal ranges, depending on the method of quantification,^2^ use of a single normal reference range has never been stipulated, rather that any reference range should be aligned with the reporting technique. In the earlier datasets derived from SSFP short‐axis imaging, manual contouring was used to delineate the endocardial border from the ventricular blood pool, resulting in an image resembling the "fjords of Norway," where papillary muscles and trabeculae were included in the left ventricular (LV) mass. A pooled meta‐analysis provided the largest reference range dataset (*n* = 288) using this "detailed" contouring technique.^3^ In contrast, in 2017 the UK Biobank project published normal reference ranges using ellipsoid "smoothed" contours of the compacted endocardial border, including 800 healthy subjects.^4^ It is unclear which of these methodologies are being used in real‐life clinical practice. To address this issue, we conducted an international survey examining contemporaneous cardiac MRI reporting practice.

An electronic survey was sent to all members of the British Society of Cardiovascular Magnetic Resonance (BSCMR) and the Australia and New Zealand Working Group for Cardiovascular Magnetic Resonance (ANZCMR). Its content was developed with reference to the SCMR guidelines for reporting cardiac MRI, and by consensus through the BSCMR and ANZCMR. Questions focused on the precise methodology and reference ranges used for assessing mass, volumes, and function.

Fifty‐five international centers participated in the study. Of the 68 adult UK centers identified, 45 (66%) returned the questionnaire; over half (58%) were from tertiary hospitals. Cardiologists completed the majority of questionnaires (82%). In Australia and New Zealand, 10 of the 12 major academic cardiac MRI centers (83%) responded.

Numerous different software vendors were used for image interpretation and postprocessing, with wide variation in the choice of normal reference ranges between centers (Table 1). For routine clinical reporting, more readers used detailed (56%) than smoothed (44%) endocardial contouring. Papillary muscles and trabeculations were included in the LV blood volume in 46%. The majority of respondents (77%) used normal reference ranges derived from methods that included papillary muscles and trabeculations as LV mass, but only 32% of observers drew them as such—a major inconsistency. Mitral valve plane tracking software was used by only 26%, while 6% did not report LV mass at all. For the right ventricle, most centers (64%) used detailed endocardial contouring, with a smaller proportion using smoothed contours (27%) or merely visual assessment (11%). Most centers (66%) did not run a specific training program on volumetric analysis.

**Table 1:** Postprocessing Software, Reference Range, and Myocardial Contouring Technique (*n* = 65)

Postprocessing software^a^	%
Circle Cvi42	56
CMRtools	5
Philips	13
Siemens Argus / Syngovia	36
Others^b^	19

Normal reference range	
Maceira et al^7^	39
Petersen et al^4^	23
Kawel‐Boehm et al^3^	17
Hudsmith et al^5^	7
Alfakih et al^6^	5
In‐house reference range/Other	9

Left Ventricular Myocardial Contours	
Detailed	56
Smoothed	44

Data are percentages taken from a total of 65 respondents from 55 international cardiac MRI centers.

a Not mutually exclusive.

b Includes QMass Medis Medical, GE Healthcare (Suiteheart), CIM (University of Auckland).

This survey has revealed wide variation between international centers in the contouring methods used to quantitate cardiac volumes, mass, and function. According to SCMR recommendations, the choice of reference ranges and clinical reporting technique should match, although these data highlight discordance. This may relate to time constraints, access to software with "thresholding" capability (ie, intensity‐based segmentation), availability of vendor‐specific reference ranges without information detailing papillary muscle inclusion/exclusion, or failure to read and understand the reference methods. It could also reflect the lack of a representative example of the contouring method in the reference article; most,^3^, ^4^, ^5^, ^6^ but not all,^7^, ^8^ published reference ranges include exemplar figures, which should be obligatory for any future reference technique publication.

We have performed volumetric and mass analyses in 20 consecutive patients (57 ± 16 years; male 70%) with hypertrophic cardiomyopathy (HCM), using the two most common postprocessing methodologies based on our survey data (Table 2).^4^, ^7^ HCM was defined by the presence of LV wall thickness ≥ 15 mm unexplained by loading conditions. Nine patients (45%) with a supranormal LV ejection fraction by detailed contours were reclassified with a normal ejection fraction by smoothed contours. Four patients (20%) with LV hypertrophy according to detailed contours had a normal indexed LV mass by smoothed contours. These data suggest using smoothed contours is untenable in HCM and its phenocopies—the presence of large papillary muscles and extensive trabeculations leads to inaccuracy, often missing hyperdynamic function.^9^, ^10^ Additionally, when serial imaging is requested to characterize the clinical course of HCM, variable contouring practice between centers will result in reported differences in LV parameters that has the potential to mislead clinicians.

**Table 2:** Comparison of Volumetric Analysis in 20 Patients With Hypertrophic Cardiomyopathy Using Detailed vs. Smoothed Contouring Methods

Parameter	Detailed contouring	Smoothed contouring	Absolute difference	Mean relative difference	*P*
LVEDV (mL)	108 ± 33	131 ± 35	+23 ± 10	+21%	<0.0001
LVESV (mL)	24 ± 13	40 ± 17	+16 ± 6	+67%	<0.01
LVSV (mL)	84 ± 25	91 ± 23	+7 ± 8	+8%	<0.001
LVEF (%)	78 ± 10	70 ± 9	−8 ± 4	−10%	<0.0001
LV mass (g)	178 ± 51	148 ± 40	−30 ± 14	−17%	<0.0001
LV mass index (g/m^2^)	90 ± 33	71 ± 17	−20 ± 26	−22%	<0.01

LV, left ventricle; EDV, end‐diastolic volume; ESV, end‐systolic volume; SV, stroke volume; EF, ejection fraction.

Data are presented as mean ± standard deviation. Comparisons were made with a two‐tailed, paired Student's *t*‐test. *P* < 0.05 was considered significant.

Inconsistent conclusions arising from analyses using these two distinct contouring techniques are not restricted to patients with overt pathology. In Fig. 1, we provide examples of myocardial contours in a healthy 52‐year‐old Caucasian female (without prior cardiovascular disease and with a normal 24‐hour ambulatory blood pressure). Again, there are important differences in the values obtained for LV parameters dependent on the contouring method. A more striking finding, however, is the difference between the two reference datasets in the normal cutoff value for LV mass. This healthy control subject is classified abnormal with eccentric LV hypertrophy according to the UK Biobank dataset,^4^ while LV mass is well within the normal range using methods described by Maceira et al.^7^

**Figure 1:**
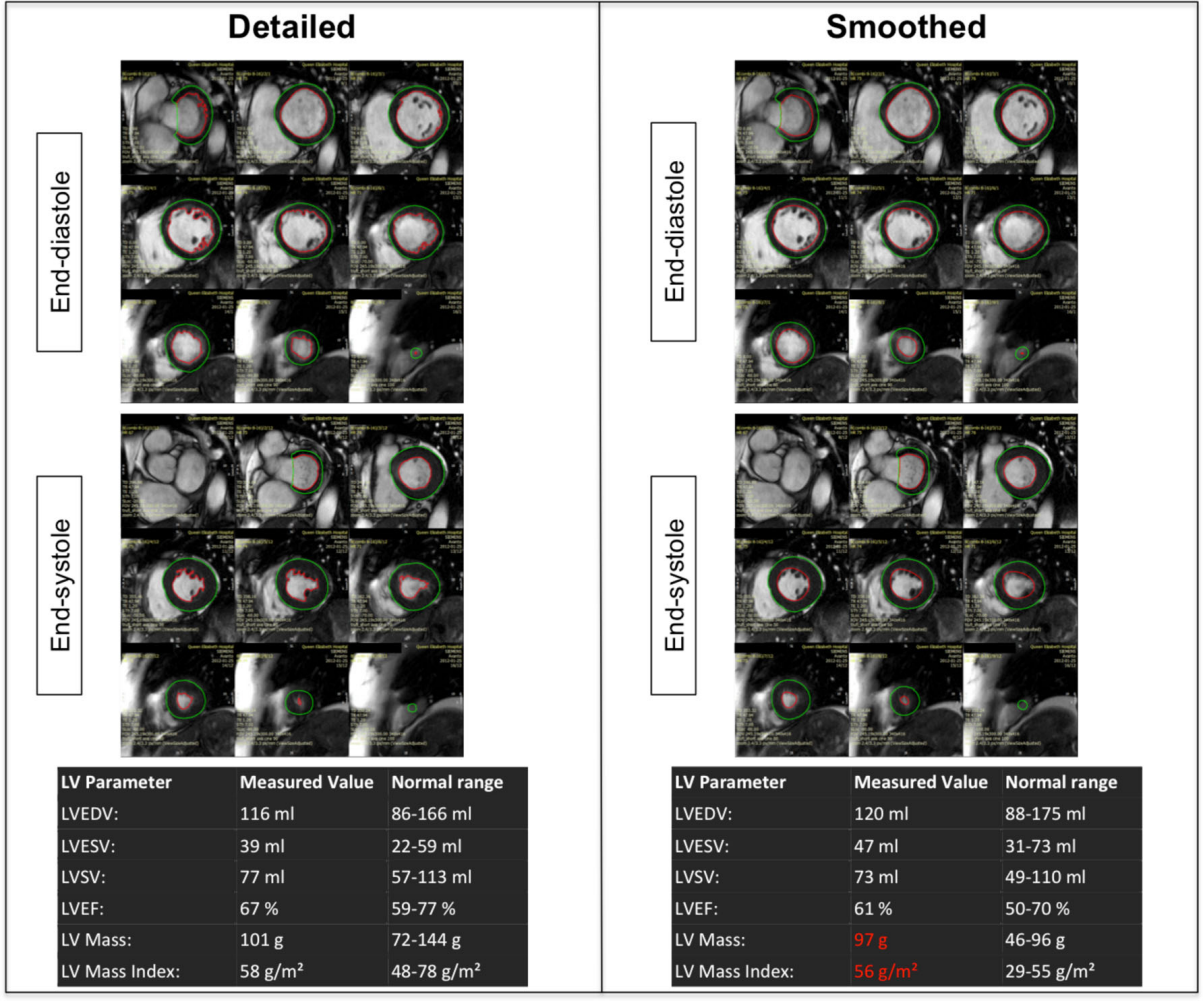
Example of detailed and smoothed left ventricular contours performed in a healthy control using reference ranges from Maceira et al^7^ and Petersen et al.^4^ The panels demonstrate contouring of the ventricles using identical SSFP cine short axis images, from base to apex at end‐diastole and end‐systole, below which are tables of the respective LV parameters. For detailed contouring, those papillary muscles and trabeculations continuous with the LV endocardial border were included in mass and excluded from the blood pool. Note not only the differences in values, but also the different classification of LV hypertrophy based on the two techniques.

The exclusion of non‐Caucasians and subjects aged < 45 years further limits the applicability of the UK Biobank normal range dataset.^4^ The authors' consensus opinion is that papillary muscles are myocardial tissue and to improve accuracy (closeness of a measured value to a true value) should routinely be excluded from blood volumes and included in LV mass. Most centers currently use software capable of producing contours using thresholding, which enables observers to perform detailed contouring that takes account of papillary muscles without sacrificing time.

The wide variation in cardiac MRI reporting practice emphasized by this survey reflects a global issue. Although machine learning holds promise (removing interobserver error, increasing standardization, and permitting reference range changes "on the fly" as models refine), its arrival into the clinical arena is not anticipated for some years. There is, therefore, a pressing need to formalize the choice of postprocessing methodology and specify a normal reference range that the MRI community should follow.

## Conflict of Interest

There are no relevant relationships with industry.

## Funding

W.E.M. was supported by a British Heart Foundation Clinical Research Fellowship Award (FS/11/17/28700).
